# Deep learning approach to femoral AVN detection in digital radiography: differentiating patients and pre-collapse stages

**DOI:** 10.1186/s12891-024-07669-7

**Published:** 2024-07-16

**Authors:** Nima Rakhshankhah, Mahdi Abbaszadeh, Atefeh Kazemi, Soroush Soltan Rezaei, Saeid Roozpeykar, Masoud Arabfard

**Affiliations:** 1 Department of Radiology and Health Research Center, Baqiyatallah University of Medical Sciences, Tehran, Iran; 2 Department of Orthopedic Surgery, Faculty of Medicine, Baqiyatallah University of Medical Sciences, Tehran, Iran; 3 Student Research Committee, Baqiyatallah University of Medical Sciences, Tehran, Iran; 4 Chemical Injuries Research Center, Systems Biology and Poisonings Institute, Baqiyatallah University of Medical Sciences, Tehran, Iran

**Keywords:** Deep learning, Osteonecrosis of the femoral head, Digital radiography, AVN detection, Artificial intelligence

## Abstract

**Objective:**

This study aimed to evaluate a new deep-learning model for diagnosing avascular necrosis of the femoral head (AVNFH) by analyzing pelvic anteroposterior digital radiography.

**Methods:**

The study sample included 1167 hips. The radiographs were independently classified into 6 stages by a radiologist using their simultaneous MRIs. After that, the radiographs were given to train and test the deep learning models of the project including SVM and ANFIS layer using the Python programming language and TensorFlow library. In the last step, the test set of hip radiographs was provided to two independent radiologists with different work experiences to compare their diagnosis performance to the deep learning models’ performance using the F1 score and Mcnemar test analysis.

**Results:**

The performance of SVM for AVNFH detection (AUC = 82.88%) was slightly higher than less experienced radiologists (79.68%) and slightly lower than experienced radiologists (88.4%) without reaching significance (p-value > 0.05). Evaluation of the performance of SVM for pre-collapse AVNFH detection with an AUC of 73.58% showed significantly higher performance than less experienced radiologists (AUC = 60.70%, p-value < 0.001). On the other hand, no significant difference is noted between experienced radiologists and SVM for pre-collapse detection. ANFIS algorithm for AVNFH detection with an AUC of 86.60% showed significantly higher performance than less experienced radiologists (AUC = 79.68%, p-value = 0.04). Although reaching less performance compared to experienced radiologists statistically not significant (AUC = 88.40%, p-value = 0.20).

**Conclusions:**

Our study has shed light on the remarkable capabilities of SVM and ANFIS as diagnostic tools for AVNFH detection in radiography. Their ability to achieve high accuracy with remarkable efficiency makes them promising candidates for early detection and intervention, ultimately contributing to improved patient outcomes.

## Introduction

Avascular necrosis of the femoral head (AVNFH) is seen in almost any age due to disturbance of blood supply to bone tissue. This blood supply disturbance may have traumatic (secondary to femoral neck fracture) or non-traumatic (chronic corticosteroid therapy, alcoholism, smoking, SLE, etc.) causes [1–3].

Considering the debilitating consequences and results of late diagnosis in the patient, timely diagnosis, and treatment of AVNFH is extremely necessary and lifesaving [4, 5]. Because of this, all the efforts of the treatment staff are aimed at diagnosing the early stages of the disease, preventing bone collapse and ultimately preventing the need for hip arthroplasty [6]. Due to the few and non-specific symptoms in the early stages of the disease and considering the overlapping of the non-specific symptoms of the disease with other causes such as transient osteoporosis of the hip, reflex sympathetic dystrophy, and subchondral stress response, it seems reasonable and valuable to use a fast, cheap and reliable method to diagnose the disease [4, 7, 8].

Hip radiography is the first imaging method for screening patients with hip pain, due to the advantages of low cost and accessibility, which is often performed as an anteroposterior (AP) view. For this reason, the simplest, cheapest, and most accessible diagnostic method, namely AP radiography of the pelvis, still maintains its importance in the whole world as a diagnostic and primary screening method for most traumatic and non-traumatic musculoskeletal problems, including AVNFH [7, 9].

According to the Ficat classification which is used in this study, AVNFH is divided into five stages with increasing severity from stage 0 (normal imaging) to stage 4 (end stage of the disease) [10]. Evaluation of these stages by doctors, especially radiologists and orthopedists need years of education and training, also considering the non-diagnosis of stage 1 of the disease with any modality, the challenge always is the accurate diagnosis of the 2nd stage and upper stages of the disease by unarmed eyes [11, 12]. In addition to the need for high experience for diagnosis, especially for 2nd stage in Ficat classification and differentiation between ARCO stage 2 and 3a [13], spending a lot of time and accuracy with the presence of human errors in the diagnosis even for the 3rd and 4th stages of the disease, due to fatigue and high workload, are the reasons for the need to develop deep learning algorithms for the AVNFH diagnosis [14, 15].

With the tremendous development of Deep Learning (DL) algorithms, Deep Convolutional Neural Networks (DCNN) have shown acceptable capabilities in disease diagnosis. By using these methods, goals such as more accurate, faster, and less costly diagnosis of diseases have become more accessible and achievable. Artificial intelligence (AI) is the ability of a machine to do tasks like human intelligence [16]. DL and DCNN are a subset of AI that uses a multilayered structure to evaluate multiple data [17]. Until today, some DCNN algorithms have helped medical doctors in different fields of orthopedics and radiology [18–20]. Unfortunately, only three studies to date have attempted to aid in the diagnosis of AVNFH using deep learning, possibly due to the nature of AVNFH itself and its diagnostic challenges for deep learning algorithms [21–23].

Our study aims to address the limitations of AVN detection in digital radiographic images through a deep learning algorithms approach and to compare the performance of deep learning and physicians.

## Materials & methods

### Study population

Our study was a retrospective study conducted in three centers under the observation of Baqiyatallah University of Medical Sciences (BMSU). The ethics committee of BMSU approved the study design. All pelvic digital radiography and MRI used in this study were extracted from the Baqiyatallah Hospital database which included patients between 2010 and 2020 years.

Patients 18 years old or older who achieved full skeletal maturity were included in this study [24]. All investigated patients included in this study had pelvic AP digital radiography and pelvic MRIs. Based on the MRI findings of pelvic AP radiography, we divided the patients into two main groups: patients with normal MRI findings, who were referred for causes of pelvic pain or other reasons other than pelvic pain, and patients with positive findings of AVNFH in pelvic MRI.

712 hips were included in the control pelvic group based on the exclusion criteria (59 hips were excluded due to long time intervals, more than 1 month, between hip radiography and hip MRI, 19 hips were excluded due to coexisting bone abnormalities such as a bone tumor, bone fracture, or orthopedic device in the femoral head and neck, 2 hips were excluded due to either poor quality MRI images or radiograph images).

Additionally, 455 hips were included in the AVNFH pelvic group based on the exclusion criteria (37 hips were excluded due to long time intervals, more than 1 month, between hip radiography and hip MRI, 31 hips were excluded due to coexisting bone abnormalities such as a bone tumor, bone fracture, or orthopedic device in the femoral head and neck, 6 hips were excluded due to either poor quality MRI images or radiograph images).

**Fig. 1:**
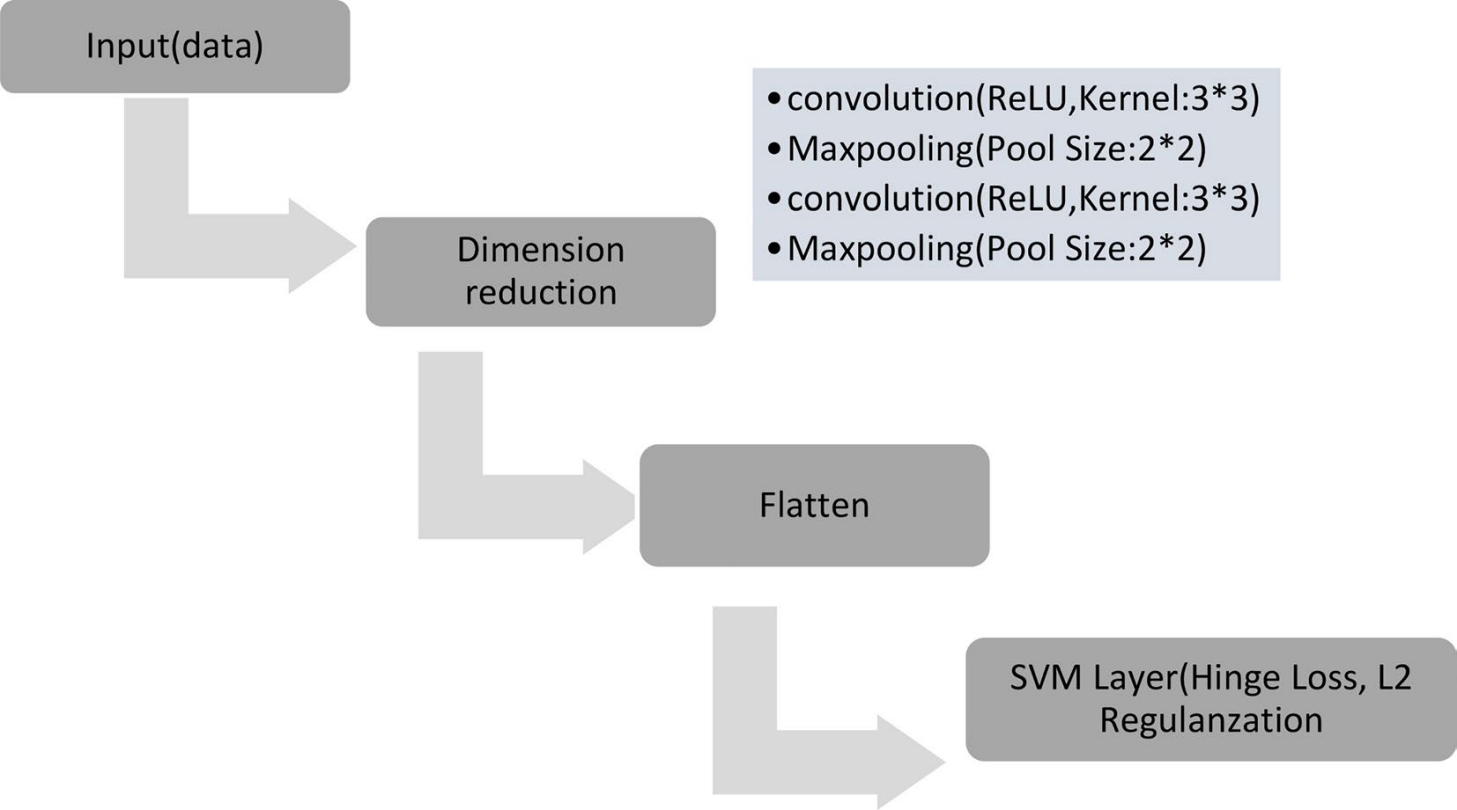
SVM model layers used in this study

In our study, we used Adaptive Neuro-Fuzzy Inference System (ANFIS) and Support vector machine (SVM) algorithms to test and train the data as additional layer in DL model. This work was done by comparing stage 0 disease with other stages (non-patient vs. patient) and comparing stage 0 disease with stage 2 disease (non-patient vs. patient with brief findings in digital radiography). According to the mentioned cases, two datasets were set up for train (*n* = 993 hips) and test (*n* = 174 hips), to examine non-patients from patients using SVM and ANFIS algorithms. Additionally, two other datasets were set up in SVM for train (*n* = 803 hips) and test (*n* = 150 hips), to examine stage 0 from stage 2 of the disease (Fig. 1).

In conclusion, the outcomes derived from the test datasets of SVM and ANFIS algorithms were juxtaposed with the outcomes procured from human resources, specifically radiologists with varying levels of experience, from less experienced to seasoned professionals.

### Digital radiography and MRI protocol

Digital pelvic radiographs were obtained by General Radiographic System - RADspeed fit (Shimadzu Healthcare). MR images were obtained by MAGNETOM Avanto eco (Siemens Healthcare) using 1.5 T imaging with the pelvic MRI protocol T1-weighted, T2-weighted, and STIR sequences at axial and coronal planes. MRI parameters for different images were as follows: Axial and coronal T1-weighted imaging (FOV, 320 × 220 mm; matrix size, 384 × 384; TR range/TE range, 650–800/10–15; slice thickness, 5.0 mm), Axial and coronal T2-weighted (FOV, 320 × 220 mm; matrix size, 384 × 384; TR range/TE range, 2000–2500/90–100; slice thickness, 5.0 mm), and coronal STIR (FOV, 320 × 220 mm; matrix size, 384 × 384; TR range/TE range, 4000–5000/50–60; TI range, 120–150; slice thickness, 5.0 mm).

### Image preprocessing

The pelvic images of the patients were cropped as left and right hips, and all images were adjusted to 250*250 pixels resolution for comparison, and the left hip images were rotated relative to the right hip for better comparison.

### Interpretation and classification of images

Two radiologists divided the patients into 5 groups based on the findings of their recent MRI images and based on the Ficat classification system.

Based on this, the pelvic radiographs were classified as follow: those who had no specific findings for AVNFH in MRI and had no problems in follow-up are classified in stage 0; those who had only bone marrow edema in MRI images and were recognized as AVNFH in the following years are in stage 1; those who have geographical lesions in the femoral head in MRI images are in stage 2; those with a crescent appearance and bone collapse in MRI are in stage 3; and finally, those with severe degenerative changes in addition to cortical bone collapse are placed in stage 4 of the disease [10].

All pelvic radiographs were divided into two separate images of the left and right hip by a radiology expert, and each hip was cut separately in the dimensions and matrix determined by the study and randomly evaluated by two independent radiologists with different work experiences. The presence or absence of AVNFH and the staging of AVNFH were assessed based on the Ficat system.

The radiologists commenting on the images and stages of the disease included a second-year radiology resident and a 10-year experienced radiologist. None of these radiologists had a role in the image preparation process, and the study was completely blinded.

### Deep learning algorithms

#### SVM-based deep learning

In the context of SVM-based deep learning, our methodological framework embraces a sophisticated approach centered around a meticulously crafted two-layer Convolutional Layer. This intricate convolutional architecture orchestrates the deployment of convolutional kernels, each infused with Rectified Linear Units (ReLU) activation, strategically adopting a kernel size of 3. Following the convolutional layers, we judiciously incorporate MaxPooling with a pool size of 2, meticulously designed to facilitate optimal dimension reduction and extraction of salient features. This thoughtful combination is underpinned by the utilization of hinge loss, a pivotal component known for fostering “maximum margin” classification, thus embodying the SVM paradigm within the realm of deep learning.

To fortify the SVM model’s adaptability and curb the risk of overfitting, we introduce L2 kernel regularization into the fray. The augmented SVM loss function (Li, reg) unfolds as a composite expression:

$$\:{L}_{i,reg}={max}\left(\text{0,1}-{y}_{i}\cdot\:\left(w\cdot\:{x}_{i}+b\right)\right)+\lambda\:\cdot\:\frac{1}{2}\cdot\:norm{\left(w\right)}^{2}$$

**Fig. 2:**
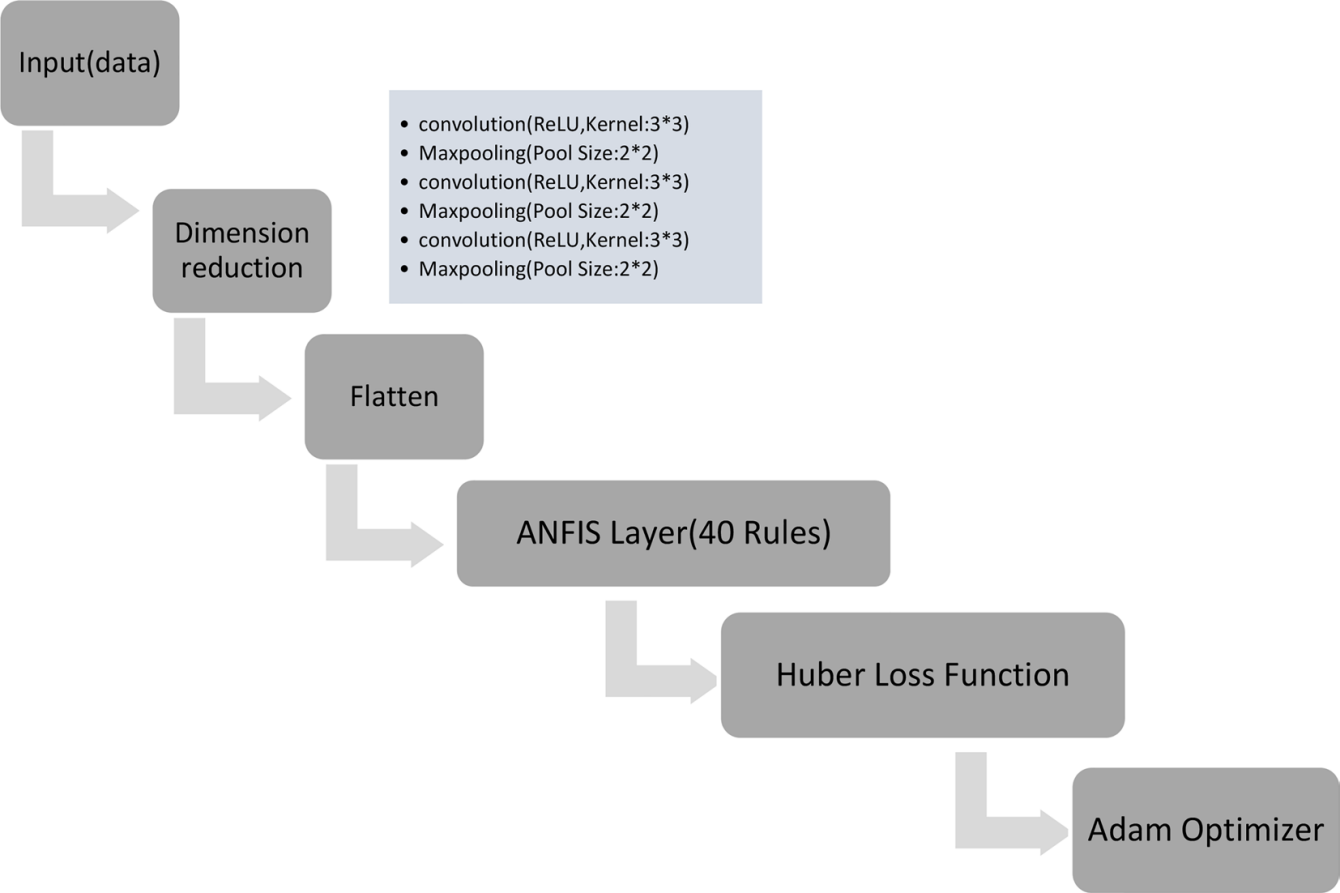
ANFIS Model layers used in this study

In this formulation, λ serves as the regularization parameter, while ∥W∥_2_ encapsulates the L2 norm of the weight matrix W. This regularization mechanism acts as a vigilant guardian, intricately navigating the delicate balance between a model that adeptly fits the data and one that avoids undue complexity. This nuanced and comprehensive approach aspires to harness the synergies between SVM and deep learning, thereby optimizing classification performance while upholding the tenets of model robustness. The entire architecture is implemented utilizing TensorFlow for seamless integration and efficient model training (Fig. 2).

#### ANFIS-based deep learning

Within the domain of deep learning featuring the ANFIS, our methodology unfolds with a nuanced approach, employing a meticulously designed five-layer Convolutional for Dimension reduction. In this intricate architecture, convolutional kernels take center stage, enriched with the dynamic activation of ReLU and a carefully chosen kernel size of 3. After the convolutional layers, a deliberate integration of MaxPooling, employing a judicious pool size of 2, serves as the cornerstone for optimal dimension reduction and the extraction of intricate features. This thoughtful architectural design not only sets the stage for the ensuing ANFIS model but also establishes a foundation for sophisticated feature representation.

The ANFIS model, a pivotal component of our methodology, boasts an elaborate configuration of 40 rules, reflecting a commitment to a nuanced and expansive inference system. Rooted in a hybrid learning approach, the ANFIS model seamlessly amalgamates the principles of fuzzy logic with the adaptive capabilities of neural networks. To facilitate robust training, the Huber loss function (LHuber) is employed, introducing a degree of resilience against the influence of outliers:

$$\:{L}_{\delta\:}\left(y,f\left(x\right)\right)=\left\{\begin{array}{c}\frac{1}{2}{\left(y-f\left(x\right)\right)}^{2}\:\:For\:\left|\begin{array}{c}y\left.-f\left(x\right)\right|\le\:\delta\:,\:\\\:otherwise.\end{array}\right.\\\:\delta\:\cdot\:(\left(\left|y\left.-f\left(x\right)\right|\right.\right)-\raisebox{1ex}{$1$}\!\left/\:\!\raisebox{-1ex}{$2$}\right.\delta\:),\end{array}\right.$$

Here, y signifies the actual output, f(x) represents the predicted output, and δ stands as the Huber loss parameter. The optimization of the ANFIS model is orchestrated through the application of the Adam optimizer, a dynamic and adaptive learning rate algorithm. This strategic choice ensures efficient convergence during the training process, accentuating the adaptability of the ANFIS model.

**Fig. 3:**
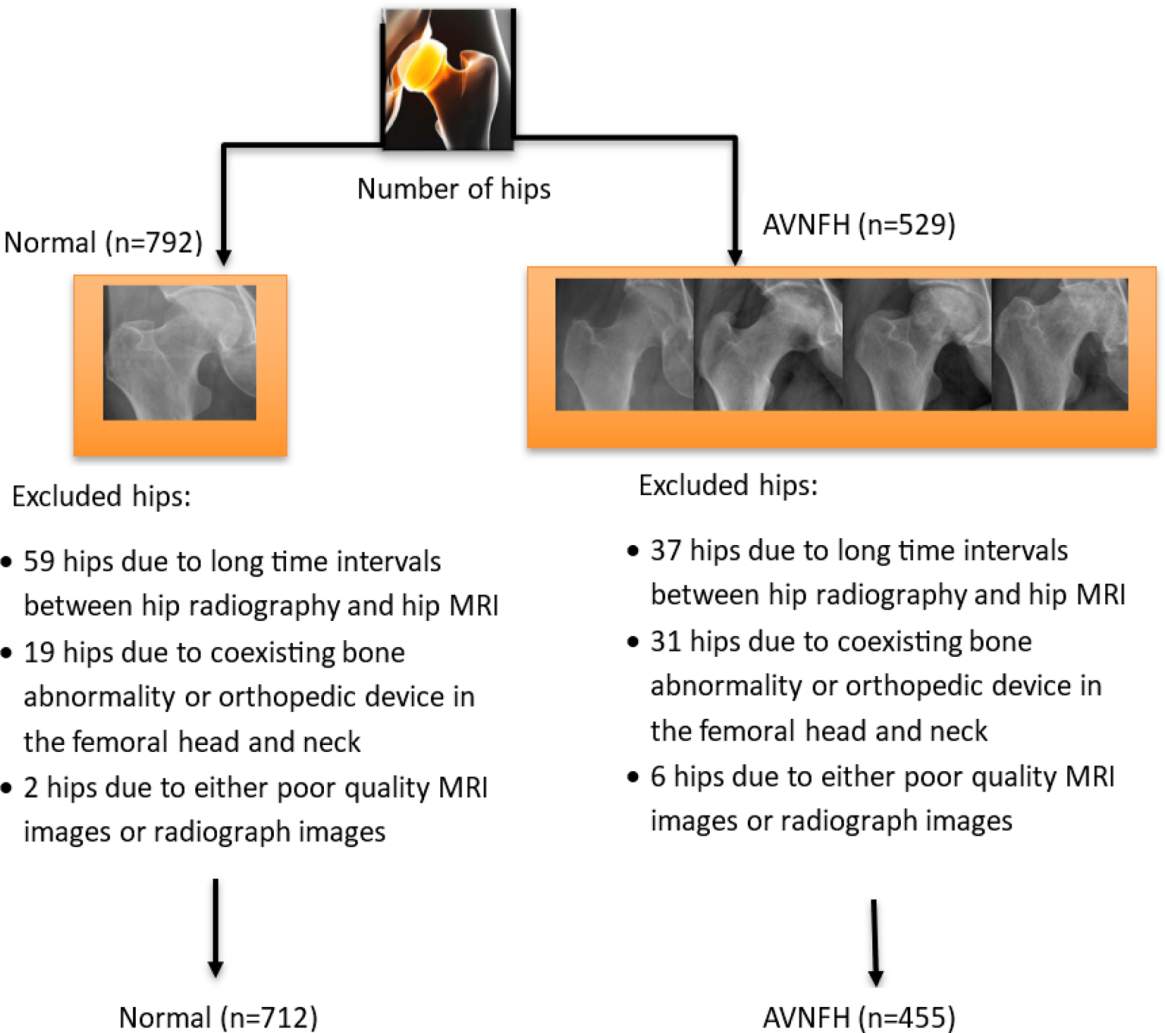
Flow chart showing study groups for analysis and machine learning

In essence, this meticulously crafted ANFIS architecture, blending the intricacies of fuzzy logic with the adaptability of neural networks, is meticulously designed to not only decipher complex patterns within the data but also mitigate the impact of outliers through the incorporation of Huber loss. The optimization process, driven by the Adam optimizer, underscores our commitment to an approach that is not only robust but also efficient in capturing the nuances of the underlying data structure (Fig. 3).

### System architecture and statistical analysis

To perform the considered deep learning model’s test and train, we first divided the patient and non-patient hips data into training and testing sets, with a ratio of 6 to 1, and analyzed the obtained data with SVM and ANFIS models (layer based DL model). From now on in the manuscript, instead of SVM or ANFIS deep learning layer model, the words SVM and ANFIS will be used in short.

In the next step, according to the importance of distinguishing the first and second stages of disease from normal hip, the data of the mentioned stages (i.e., stages 0 to 2) are separated independently with a ratio of 6 to 1 into training and testing sets, to check the performance of the SVM model. In the training phase, we performed 10-fold cross-validation (CV) to validate the models’ performance on the training and testing sets.

Demographic findings were evaluated by descriptive statistics and presented as numbers and mean ± standard deviation (SD). The non-parametric Mann–Whitney U test was used to compare the ages of patients between the two groups. Sensitivity, specificity, positive predictive value (PPV) and negative predictive value (NPV), accuracy, and AUC were calculated for each model performance and radiologist.

Radiologists and model performance comparison was achieved with the use of receiver operating characteristics (ROC) curves and the area under the curve (AUC) with 95% confidence intervals calculated with bootstrapping using the pROC package [25]. A single threshold value at 0.5 was used for the ROC curves given the fact that upon augmentation groups were balanced. Comparisons between the AUCs of the models and readers were performed with DeLong’s test in the IBM SPSS Statistics 27.0.1 software. Significance was defined with a p-value lower than 0.05 in all analyses.

## Results

### Patient demographics

In the study conducted, after applying the mentioned exclusion criteria and removing inappropriate hip digital radiographs, a total of 1167 hips were included in the analysis: 455 hips with AVNFH and 712 hips without AVNFH were included in the study. The average age of the group without AVNFH was equal to 44.6 ± 12.5 and the group with AVNFH was equal to 46.7 ± 12.7, and there was no statistically significant difference between the ages of the noted study groups (p-value = 0.437) (Table 1)(Fig. 1). Additionally, no gender difference was observed between the groups.

**Table 1:** Demographic information of patients

Characteristic	Number of hips	Age (y), mean ± SD	Comparison of stage 0 with stage 2 in SVM^1^		Comparison of stage 0 with other stages in SVM^1^		Comparison of stage 0 with other stages in ANFIS^2^	
			training set	testing set	training set	testing set	training set	testing set
Normal group (stage 0)	712	44.60 ± 12.50	606	106	606	106	606	106
AVNFH group	455	46.70 ± 12.70	197	34	387	68	387	68
stage 1&2	231	43.60 ± 12.50	197	34	197	34	197	34
stage 3	140	49.20 ± 14.60	NA	NA	120	20	120	20
stage 4	84	51.20 ± 10.00	NA	NA	70	14	70	14

1: Support vector machine, 2: Adaptive Neuro-Fuzzy Inference System, *NA: not appliable*

### Radiomics analysis and machine learning model performance

**Fig. 4:**
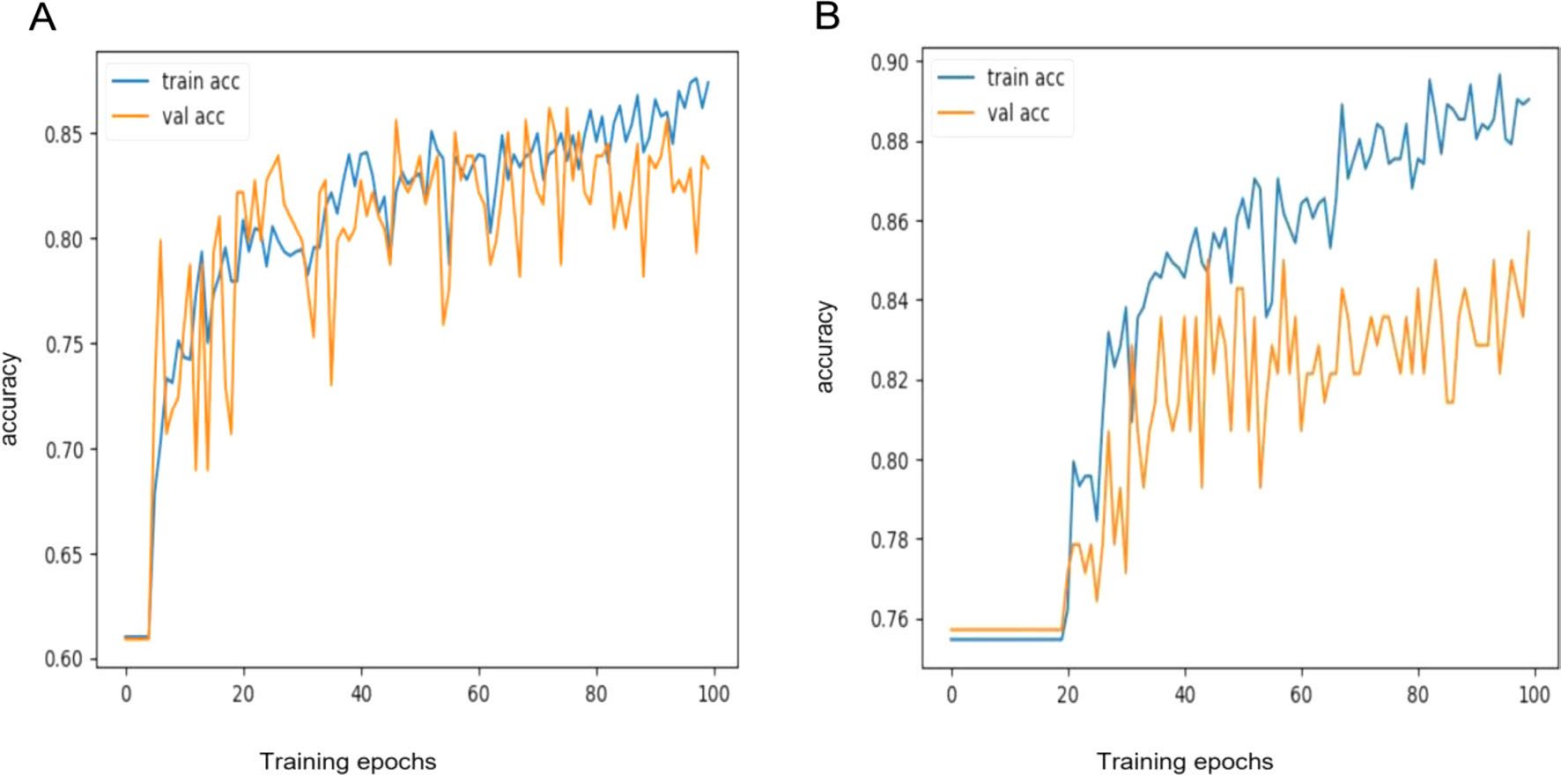
The behavior observed during the training of SVM model (100 epochs of SVM training). **A**: Changes in accuracy during training in both the validation and training sets comparing between stage 0 with other stages (non-patient vs. patient). **B**: Changes in accuracy during training in both the validation and training sets comparing stage 0 with stage 2(non-patient vs. patient with brief findings in digital radiography)

Following data preprocessing and scaling, a dataset of 993 hips (387 AVNFH hips and 606 normal hips) was divided for subsequent DL models training, In the training set, the SVM algorithm exhibited an accuracy of 86.21% (80.18–90.96%) over 100 epochs (Fig. 4). The operating point of the SVM algorithm was set to achieve accuracy for detecting AVNFH. At the highest achieved accuracy, the performance of the test set was checked for the SVM algorithm. The Sensitivity and specificity for the SVM algorithm in detecting AVNFH from normal hips were 67.65% (55.21–78.49%CI) and 98.11% (93.35–99.77%CI), respectively. Additionally, AUC (95% CI) for the SVM algorithm was obtained as 82.88% (74.21–88.13%). This threshold was used throughout subsequent analyses (Table 2).

**Table 2:** Diagnostic performance of Deep Learning (DL) algorithms for avascular necrosis of femoral head (AVNFH) in digital radiography

Performance Measure	SVM^1^ (Comparison of stage 0 with stage 2)	SVM^1^ (Comparison of stage 0 with other stages)	ANFIS^2^ (Comparison of stage 0 with other stages)
AUC^*^ (95% CI)	73.58% (62.30–83.19%)	82.88% (74.21–88.13%)	86.60% (77.80–93%)
Sensitivity	50.00% (32.43–67.57%)	67.65% (55.21–78.49%)	77.05% (64.50–86.85%)
Specificity	97.17% (91.95–99.41%)	98.11% (93.35–99.77%)	96.46% (91.18–99.03%)
PPV^**^	85.00% (63.87–94.78%)	95.83% (85.23–98.92%)	92.16% (81.63–96.88%)
NPV^***^	85.83% (81.21–89.47%)	82.54% (77.01–86.97%)	88.62% (83.08–92.51%)
Accuracy	85.71% (78.80–91.05%)	86.21% (80.18–90.96%)	89.66% (84.14–93.75%)

Note—Data in parentheses are 95% Confidence intervals

1: Support vector machine, 2: Adaptive Neuro-Fuzzy Inference System

* : Area under the curve, **: Positive Predictive Value, ***: Negative Predictive Value

**Fig. 5:**
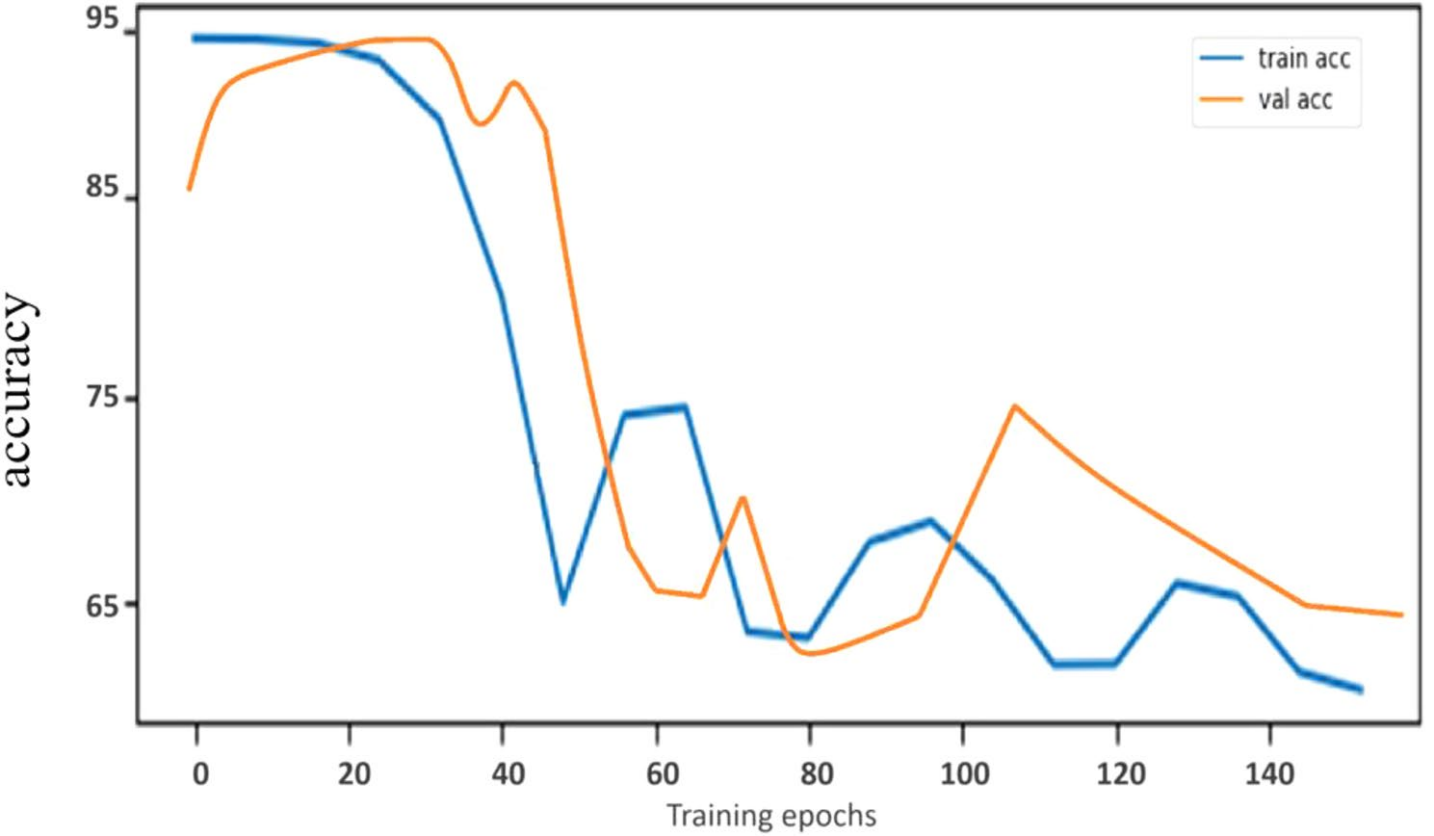
The behavior observed during the training of ANFIS model ( 140 epochs of ANFIS training). Changes in accuracy during training in both the validation and training sets comparing stage 0 to other stages (non-patient vs. patient)

The ANFIS algorithm surpassed the SVM algorithm with 89.66% (84.14–93.75%CI) accuracy in just 20 epochs, highlighting its superior efficiency in reaching convergence (Fig. 5). At the highest achieved accuracy, the performance of the test set was checked for the ANFIS algorithm. The Sensitivity and specificity for the ANFIS algorithm in detecting AVNFH from normal hips were 77.05% (64.50–86.85%CI) and 96.46% (91.18–99.03%CI), respectively. Additionally, AUC (95% CI) for the ANFIS algorithm was obtained as 86.6% (77.8–93%). This threshold was used throughout subsequent analyses (Table 2).

In the normal hip/pre-collapse AVFNH classification (comparison of stage 0 with stage 1&2) task, the SVM algorithm attained 85.71% (78.80–91.05%CI) accuracy in 100 epochs. The Sensitivity and specificity for the SVM algorithm in detecting pre-collapse were 50% (32.43–67.57%CI) and 97.17% (91.95–99.41%CI), respectively. Additionally, AUC (95% CI) for the SVM algorithm was obtained as 73.58% (62.30–83.19%). Unfortunately, the ANFIS algorithm did not show acceptable performance for the normal hip/pre-collapse AVFNH classification task (Table 2).

### Comparison of machine learning algorithms to radiologists

In this step, the data that was separated in the previous step as a test set for DL algorithms was given to two radiologists with different work experiences for review, and each radiologist gave his opinion about the set of hip radiographs. In the subset of AVNFH detection (other stages in comparison to stage 0), the less experienced radiologist achieved a sensitivity, specificity, and AUC (95% CI) of 73.53% (61.43–83.50%CI), 85.85% (77.74–91.86%CI) and 79.68% (69.6–87.68%CI), respectively. Whereas the experienced radiologist’s sensitivity, specificity, and AUC (95% CI) for AVNFH detection was 85.29% (74.61–92.72%CI), 91.51% (84.49–96.04%CI), and 88.4% (79.20–94.12%CI), respectively. In the subset of pre-collapse AVNFH detection (stage 1&2 in comparison to stage 0), the less experienced radiologist achieved sensitivity, specificity, and AUC (95% CI) of 41.18% (24.65–59.30%CI),80.19% (71.32–87.30%CI), 60.7% (48–73.30%CI), respectively. Whereas the experienced radiologist sensitivity, specificity, and AUC (95% CI) for detection of pre-collapse AVNFH was 61.76% (43.56–77.83%CI), 84.91% (76.65–91.12%CI) and 73.33% (60.20–84.40%CI), respectively (Table 3).

**Table 3:** Diagnostic performance of radiologists for avascular necrosis of femoral head (AVNFH) in digital radiography

Performance Measure	Less Experienced A (Comparison of stage 0 with stage 2)	Experienced B (Comparison of stage 0 with stage 2)	Less Experienced A (Comparison of stage 0 with other stages)	Experienced B (Comparison of stage 0 with other stages)
AUC * (95% CI)	60.70% (48–73.30%)	73.33% (60.20–84.40%)	79.68% (69.60–87.68%)	88.4% (79.20–94.12%)
Sensitivity	41.18% (24.65–59.30%)	61.76% (43.56–77.83%)	73.53% (61.43–83.50%)	85.29% (74.61–92.72%)
Specificity	80.19% (71.32–87.30%)	84.91% (76.65–91.12%)	85.85% (77.74–91.86%)	91.51% (84.49–96.04%)
PPV**	40.00% (27.68–53.73%)	56.76% (43.75–68.89%)	76.92% (67.13–84.48%)	86.57% (77.39–92.39%)
NPV***	80.95% (75.95–85.11%)	87.38% (81.76–91.45%)	83.49% (77.15–88.33%)	90.65% (84.51–94.52%)
Accuracy	70.71% (62.43–78.09%)	79.29% (71.62–85.67%)	81.03% (74.41–86.57%)	89.08% (83.47–93.30%)

Note—Data in parentheses are 95% Confidence intervals

A: second-year radiology resident, B: 10-year experienced radiologist

* :Area under the curve, **: Positive Predictive Value, ***: Negative Predictive Value

AUC (95% CI) prepared by deep learning algorithms and radiologists were compared with statistically significant test results of p-value ≤ 0.05 using DeLong’s test. The performance of SVM algorithm for AVNFH detection was slightly higher than less experienced radiologist and slightly lower than experienced radiologist without reaching significance (p-value > 0.05) (Table 4). Evaluation of the performance of SVM for pre-collapse AVNFH detection with the AUC of 73.58% showed significantly higher performance than less experienced radiologists (AUC = 60.70%, p-value < 0.001). On the other hand, no significant difference is noted between experienced radiologist and SVM for pre-collapse detection (Table 4).

**Table 4:** Comparison of SVM algorithm to radiologists

	SVM^1^ algorithm	Less Experienced ^A^	Experienced ^B^
AVNFH detection (stage 0 from other stages) AUC * (%95 CI)	82.88%	79.68%	88.4%
p-value ^**^		0.53	0.10
PRE-COLLAPSE AVNFH detection (stage 0 from stage 2) AUC ^*^ (%95 CI)	73.58%	60.70%	73.33%
p-value		< 0.001^***^	0.30

A: second-year radiology resident, B: 10-year experienced radiologist

1: Support vector machine

DeLong’s test p-values on AUC between SVM and radiologists

^*^: Area under the curve, **: p-value of the comparison of each reader to SVM; ***: statistically significant value

The ANFIS algorithm for AVNFH detection with the AUC of 86.60% (77.80–93%CI) showed significantly higher performance than less experienced radiologist (AUC = 79.68% (69.60–87.68%CI), p-value = 0.04). However, although the ANFIS algorithm’s performance was slightly lower than the experienced radiologist (AUC = 88.40%), the difference was not statistically significant (p-value = 0.2) (Table 5).

**Table 5:** Comparison of ANFIS algorithm to radiologists

	ANFIS1 algorithm	Less Experienced A	Experienced B
AVNFH detection (stage 0 from other stages) AUC* (%95 CI)	86.60%	79.68%	88.40%
p-value^**^		0.04^***^	0.2

A: second-year radiology resident, B: 10-year experienced radiologist

1: Adaptive Neuro-Fuzzy Inference System

DeLong’s test p-values on AUC between ANFIS and radiologists

^*^: Area under the curve, **: p-value of the comparison of each reader to ANFIS; ***: statistically significant value

## Discussion

Radiography is the first diagnostic method in patients with pelvic pain and pelvic trauma. Different studies have been conducted in the field of pelvic radiography by DL. Some studies have been carried out to investigate the angles and sizes of the femur bone with artificial intelligence for surgical purposes and have been able to achieve favorable results [26–29].

Other studies have moved in the direction of diagnosis and have mainly used DL algorithms to investigate bone fractures of the femoral head and neck [30–32]. In the meantime, we can refer to the study of Hsieh and his colleagues who, with a DL model called DAFDNet, were able to achieve 94.8% accuracy in detecting femoral neck fractures without displacement, which is a better performance compared to older algorithms such as Densenet and U-net [30]. In another study conducted by Liu and his colleagues on the diagnosis of femoral intertrochanteric fracture, the DL algorithm, Faster-RCNN was able to achieve an accuracy of 88% more than the diagnostic accuracy of orthopedists [31]. In one study based on AVN, Wernér et al. used a segmentation-based DL model to diagnose lunate AVN. They achieved a sensitivity of 93.33%, specificity of 93.28%, accuracy of 93.28%, and AUC of 0.94% (95% 0.88–0.99 CI), which had better results than one expert and lower results than another expert [33].

Despite the mentioned studies about femoral head and neck with different algorithms, only a few studies have investigated AVNFH disease and its differentiation from normal cases and other causes [21–23]. This is while the early and timely diagnosis of AVNFH is extremely beneficial to the patient and prevents the consequences of late diagnosis such as femoral head collapse and the need for surgery [34].

However, it is difficult to diagnose or suspect the disease with the unaided eye of a doctor, especially during the pre-collapse stages of the disease. In this situation, doctors take two conservative and non-conservative approaches in dealing with pelvic pain, the first approach leads to unnecessary pelvic MRIs in most people, and the second approach, is based on denying patients’ symptoms and referring to the symptoms as psychosomatic symptoms, sometimes leads to missing the early stages of the disease [35, 36].

In our study, we developed and trained two DL models, SVM and ANFIS, that could predict AVNFH in digital radiography. When deciding between ANFIS, SVM, and ANN for image analysis, consider the strengths each model offers. ANFIS excels in handling complex, non-linear relationships and uncertainties through its fuzzy logic, making it suitable for scenarios where data patterns are intricate and difficult to discern. SVM, on the other hand, is effective in dealing with high-dimensional data and can work well with smaller training datasets, making it a good choice for resource-constrained environments. These models also offer interpretability, with ANFIS providing insights through fuzzy rules and SVM offering clear decision boundaries. These strengths make ANFIS and SVM valuable options when analyzing radiology images, especially when computational resources are limited or when interpretability is crucial.

Our study showed that the performance of both DL models (SVM & ANFIS) in detecting AVNFH is superior to the less experienced radiologist in the detection of AVNFH without statistical significance. These findings are very similar to Li and his colleagues’ study which used the proposed AVN-Net algorithm to detect AVNFH with the F1 score of 0.9242 [21]. Additionally, similar findings were obtained in the study of Chee and his colleagues in the ability to diagnose AVNFH disease in radiography with sensitivity and specificity of 75.2% and 97.2%, respectively [22]. In another study based on MRI AVNFH detection, Klontzas et al. showed similar AVNFH detection performance as their proposed CNN (AUC of 85.50%) compared to two MSK experts (the first expert achieved an AUC of 75.70%, whereas the second achieved an AUC of 73.08%) without a significant difference [23].

Although there is no significant difference in the ability of DL models and radiologists to distinguish patients from non-patients, it should be noted that the increased workload of radiologists leads to a significant loss of diagnostic power [14]. For this reason, it seems that the use of deep learning is more reasonable both in terms of reducing diagnosis time and in terms of reducing diagnostic errors [19, 21, 37].

Considering the importance of differentiating the pre-collapse stage from the absence of disease (stage 2 in comparison to stage 0) and the existence of high human error in differentiating these stages [14, 38], our study compared the ability of DL models and radiologists to differentiate these states.

The SVM model surprisingly showed a significantly better performance in diagnosing stage 2 than stage 0 compared to the radiology resident with a statistically significant p-value of less than 0.05, which can lead to using the DL models as auxiliary tools in teaching hospitals in the future. In examining the performance of SVM in diagnosing stage 2 from stage 0, no significant difference was seen compared to experienced radiologists, which shows that the DL model can be used in areas where there is no access to experienced radiologists.

It should be noted that the ANFIS model did not perform convincingly in differentiating stage 2 from stage 0, and the results were not acceptable.

The superior performance demonstrated by both SVM and ANFIS algorithms indicates their substantial potential as viable diagnostic instruments for facilitating early detection and intervention. These findings, while promising, underscore the necessity for a more comprehensive understanding of the operational mechanisms that underlie these algorithms. A detailed examination will aid in enhancing their efficacy and reliability, thereby ensuring their optimal performance in clinical predictions and decision-making.

Furthermore, this research serves as a compelling impetus for additional investigation into the algorithms’ wider applications in the medical field. A broader adoption of these algorithms in clinical settings could potentially revolutionize diagnostic procedures, particularly in challenging domains where human expertise is limited or where the speed of diagnosis is critical. Longitudinal studies are recommended to evaluate the performance of these algorithms over extended periods. This would provide invaluable insights into their stability, consistency, and adaptability in response to evolving medical data and shifting patient demographics.

## Conclusion

The transition from theoretical validation to practical application will require the establishment of rigorous validation protocols and ethical guidelines, to ensure the responsible and equitable use of these sophisticated diagnostic tools. Hence, this necessitates a close collaboration between researchers, clinicians, ethicists, and policymakers, aiming for a holistic integration of these algorithms into the healthcare system, while addressing potential challenges and risks.

In conclusion, our study has shed light on the remarkable capabilities of SVM and ANFIS as diagnostic tools for AVNFH detection in radiography. Their ability to achieve high accuracy with remarkable efficiency makes them promising candidates for early detection and intervention, ultimately contributing to improved patient outcomes.

## Data Availability

The datasets used during the current study are available from the corresponding author upon reasonable request.

## References

[1] Mankin HJ (1992). Nontraumatic necrosis of bone (osteonecrosis). N Engl J Med.

[2] Aldridge JM 3rd, Urbaniak JR. Avascular necrosis of the femoral head: etiology, pathophysiology, classification, and current treatment guidelines. Am J Orthop (Belle Mead NJ). 2004;33(7):327–32. 15344574

[3] Soucacos PN, Urbaniak JR (2004). Osteonecrosis of the human skeleton. Orthop Clin North Am.

[4] Hernigou P, Poignard A, Nogier A, Manicom O (2004). Fate of very small asymptomatic stage-I osteonecrotic lesions of the hip. J Bone Joint Surg Am.

[5] Nam KW, Kim YL, Yoo JJ, Koo KH, Yoon KS, Kim HJ (2008). The fate of untreated asymptomatic osteonecrosis of the femoral head. J Bone Joint Surg Am.

[6] Nishii T, Sugano N, Ohzono K, Sakai T, Haraguchi K, Yoshikawa H. Progression and cessation of collapse in osteonecrosis of the femoral head. Clin Orthop Relat Res. 2002(400):149–57. 10.1097/00003086-200207000-00019 12072757

[7] Konarski W, Poboży T, Śliwczyński A, Kotela I, Krakowiak J, Hordowicz M, et al. Avascular Necrosis of Femoral Head&mdash; Overview and Current State of the Art. International Journal of Environmental Research and Public Health. 2022;19(12):7348. 10.3390/ijerph19127348 PMC9223442 35742595

[8] Moya-Angeler J, Gianakos AL, Villa JC, Ni A, Lane JM (2015). Current concepts on osteonecrosis of the femoral head. World J Orthop.

[9] Manenti G, Altobelli S, Pugliese L, Tarantino U (2015). The role of imaging in diagnosis and management of femoral head avascular necrosis. Clin Cases Min Bone Metab.

[10] Jawad MU, Haleem AA, Scully SP (2012). In brief: Ficat classification: avascular necrosis of the femoral head. Clin Orthop Relat Res.

[11] Stöve J, Riederle F, Kessler S, Puhl W, Günther KP (2001). [Reproducibility of radiological classification criteria of femur head necrosis]. Z Orthop Ihre Grenzgeb.

[12] Kay RM, Lieberman JR, Dorey FJ, Seeger LL. Inter- and intraobserver variation in staging patients with proven avascular necrosis of the hip. Clin Orthop Relat Res. 1994(307):124–9. 7924024

[13] Zhao D, Zhang F, Wang B, Liu B, Li L, Kim SY (2020). Guidelines for clinical diagnosis and treatment of osteonecrosis of the femoral head in adults (2019 version). J Orthop Translat.

[14] Berlin L (2000). Liability of interpreting too many radiographs. AJR Am J Roentgenol.

[15] Alpert HR, Hillman BJ (2004). Quality and variability in diagnostic radiology. J Am Coll Radiol.

[16] Amasya H, Yildirim D, Aydogan T, Kemaloglu N, Orhan K (2020). Cervical vertebral maturation assessment on lateral cephalometric radiographs using artificial intelligence: comparison of machine learning classifier models. Dentomaxillofac Radiol.

[17] LeCun Y, Bengio Y, Hinton G (2015). Deep learning. Nature.

[18] Gale W, Oakden-Rayner L, Carneiro G, Bradley AP, Palmer LJ. Detecting hip fractures with radiologist-level performance using deep neural networks. ArXiv. 2017; abs/1711.06504.

[19] Urakawa T, Tanaka Y, Goto S, Matsuzawa H, Watanabe K, Endo N (2019). Detecting intertrochanteric hip fractures with orthopedist-level accuracy using a deep convolutional neural network. Skeletal Radiol.

[20] Klontzas ME, Manikis GC, Nikiforaki K, Vassalou EE, Spanakis K, Stathis I et al. Radiomics and Machine Learning can differentiate transient osteoporosis from avascular necrosis of the hip. Diagnostics (Basel). 2021;11(9). 10.3390/diagnostics11091686 PMC8468167 34574027

[21] Li Y, Li Y, Tian H (2021). Deep learning-based end-to-end diagnosis system for avascular necrosis of femoral head. IEEE J Biomed Health Inf.

[22] Chee CG, Cho J, Kang Y, Kim Y, Lee E, Lee JW (2019). Diagnostic accuracy of digital radiography for the diagnosis of osteonecrosis of the femoral head revisited. Acta Radiol.

[23] Klontzas ME, Vassalou EE, Spanakis K, Meurer F, Woertler K, Zibis A (2024). Deep learning enables the differentiation between the early and late stages of hip avascular necrosis. Eur Radiol.

[24] Nahhas RW, Sherwood RJ, Chumlea WC, Towne B, Duren DL (2013). Predicting the timing of maturational spurts in skeletal age. Am J Phys Anthropol.

[25] Robin X, Turck N, Hainard A, Tiberti N, Lisacek F, Sanchez JC (2011). pROC: an open-source package for R and S + to analyze and compare ROC curves. BMC Bioinformatics.

[26] Larson N, Nguyen C, Do B, Kaul A, Larson A, Wang S (2022). Artificial Intelligence System for Automatic Quantitative Analysis and Radiology Reporting of Leg length radiographs. J Digit Imaging.

[27] Archer H, Reine S, Alshaikhsalama A, Wells J, Kohli A, Vazquez L (2022). Artificial intelligence-generated hip radiological measurements are fast and adequate for reliable assessment of hip dysplasia: an external validation study. Bone Jt Open.

[28] Moon K-R, Lee B-D, Lee MS (2023). A deep learning approach for fully automated measurements of lower extremity alignment in radiographic images. Sci Rep.

[29] Rouzrokh P, Wyles CC, Kurian SJ, Ramazanian T, Cai JC, Huang Q et al. Deep Learning for Radiographic Measurement of Femoral Component Subsidence Following Total Hip Arthroplasty. Radiology: Artificial Intelligence. 2022;4(3):e210206. 10.1148/ryai.210206 PMC9152683 35652119

[30] Hsieh SL, Chiang JL, Chuang CH, Chen YY, Hsu CJ. A computer-assisted diagnostic method for Accurate detection of early nondisplaced fractures of the femoral Neck. Biomedicines. 2023;11(11). 10.3390/biomedicines11113100 PMC10669449 38002100

[31] Liu P, Lu L, Chen Y, Huo T, Xue M, Wang H (2022). Artificial intelligence to detect the femoral intertrochanteric fracture: the arrival of the intelligent-medicine era. Front Bioeng Biotechnol.

[32] Twinprai N, Boonrod A, Boonrod A, Chindaprasirt J, Sirithanaphol W, Chindaprasirt P (2022). Artificial intelligence (AI) vs. human in hip fracture detection. Heliyon.

[33] Wernér K, Anttila T, Hulkkonen S, Viljakka T, Haapamäki V, Ryhänen J (2024). Detecting avascular necrosis of the lunate from radiographs using a deep-learning model. J Imaging Inf Med.

[34] Ando W, Yamamoto K, Koyama T, Hashimoto Y, Tsujimoto T, Ohzono K (2017). Radiologic and clinical features of misdiagnosed idiopathic osteonecrosis of the femoral head. Orthopedics.

[35] Baiguissova D, Laghi A, Rakhimbekova A, Fakhradiyev I, Mukhamejanova A, Battalova G (2023). An economic impact of incorrect referrals for MRI and CT scans: a retrospective analysis. Health Sci Rep.

[36] Karantanas AH (2013). Accuracy and limitations of diagnostic methods for avascular necrosis of the hip. Expert Opin Med Diagn.

[37] Liu FY, Chen CC, Cheng CT, Wu CT, Hsu CP, Fu CY et al. Automatic hip detection in Anteroposterior pelvic Radiographs-A Labelless practical Framework. J Pers Med. 2021;11(6). 10.3390/jpm11060522 PMC8226859 34200151

[38] Fitzgerald R (2001). Error in radiology. Clin Radiol.

